# Interleaving cortex-analog mixing improves deep non-negative matrix factorization networks

**DOI:** 10.3389/fncom.2025.1692418

**Published:** 2025-11-05

**Authors:** Mahbod Nouri, David Rotermund, Alberto Garcia-Ortiz, Klaus R. Pawelzik

**Affiliations:** ^1^Institute for Theoretical Physics, University of Bremen, Bremen, Germany; ^2^Institute of Electrodynamics and Microelectronics (ITEM.ids), University of Bremen, Bremen, Germany

**Keywords:** deep neuronal networks, non-negative matrix factorization (NMF), back-propagation error learning, cortical column, convolutional neural networks (CNN)

## Abstract

Considering biological constraints in artificial neural networks has led to dramatic improvements in performance. Nevertheless, to date, the positivity of long-range signals in the cortex has not been shown to yield improvements. While Non-negative matrix factorization (NMF) captures biological constraints of positive long-range interactions, deep convolutional neural networks with NMF modules do not match the performance of conventional neural networks (CNNs) of a similar size. This work shows that introducing intermediate modules that combine the NMF's positive activities, analogous to the processing in cortical columns, leads to improved performance on benchmark data that exceeds that of vanilla deep convolutional networks. This demonstrates that including positive long-range signaling together with local interactions of both signs in analogy to cortical hyper-columns has the potential to enhance the performance of deep networks.

## 1 Introduction

The success of modern neural networks has often come from incorporating principles inspired by their biological role model: the brain. A well-known example is the introduction of convolutional layers (Lecun et al., 1998), which mirror the organization of the visual cortex in biological brains. Neurons in the visual cortex have localized receptive fields, meaning they respond stereotypically to stimuli in specific regions of the visual field (Hubel and Wiesel, 1962). This principle, first adopted by Fukushima (1980), now enables convolutional neural networks (CNNs) to efficiently detect patterns and hierarchies in images. This biologically inspired design has been crucial in advancing the performance of machine learning models in computer vision (Lecun et al., 1998).

Despite the success of incorporating some biological principles, several key constraints in biological neural systems remain underexplored in machine learning. For example, Dale's Law, one of the core principles of neurobiology, dictates that neurons are either excitatory or inhibitory but not both (Strata and Harvey, 1999).

Another often overlooked biological aspect is the nature of long-range connections between cortical areas, such as those between the primary visual cortex (V1) and higher visual areas. In biological neural systems, long-range connections are predominantly excitatory, in contradiction with usual deep CNNs. In the cortex, the excitatory postsynaptic currents (EPSCs) are also received by inhibitory neurons, which only locally modulate pyramidal (Pyr) cells through inhibitory postsynaptic currents (IPSCs) (Yang et al., 2013), typically in a different layer. This configuration implies that while interactions of both signs are essential for modulating neural responses and shaping information processing locally, the primary flow of information across layers and areas is governed by excitatory or "positive" connections.

Our work also builds on the hypothesis that receptive fields in the visual cortex develop through sparse coding mechanisms—where neural activity is distributed such that only a small subset of neurons responds to a given stimulus (Olshausen and Field, 2006). Non-negative matrix factorization (NMF) (Lee and Seung, 1999, 2000) provides an elegant mathematical framework that simultaneously satisfies two key biological constraints: the positivity of long-range neural interactions and the tendency toward sparse representations. For instance, Hoyer (2003) demonstrated that such sparse coding mechanisms could be effectively modeled using NMF. By imposing both non-negativity and sparseness constraints, this work showed that NMF can learn parts-based, interpretable representations similar to the mechanism yielding receptive fields first proposed in Olshausen and Field (1996).

While Non-negative Matrix Factorization (NMF) is a powerful unsupervised learning technique used in various fields, including signal processing, computer vision, and data mining (Lee and Seung, 1999), applying it effectively to supervised tasks like computer vision presents significant challenges. When used in isolation, NMF typically cannot match the performance of modern deep learning architectures such as CNNs. Previous works have attempted to bridge this gap by combining NMF with deep learning approaches.

For instance, Geng et al. (2021) proposed using an NMF layer on top of a convolutional model. However, such implementations often reinitialize and retrain the NMF components from scratch after each forward pass, leading to computational inefficiency and potential instability. In contrast, our approach implements a hierarchical NMF architecture where the NMF weights are treated as learnable parameters and optimized through back-propagation alongside the network's other parameters. This enables the NMF components to adapt continuously to the task requirements while maintaining their biological constraints. Missing in current networks using NMF are local interactions that include inhibition, an important property of cortical microcircuits (Martin, 1991; Callaway, 1998).

After briefly reviewing NMF, we introduce a convolutional network architecture where we exchange CNN modules with NMF modules. We then propose a simple but novel extension of the NMF network where subsequent 1 × 1 convolutional layers are inserted. Thereby, we realize general local interactions among the features of the NMF modules in analogy to cortical hyper-columns, which makes these networks a step toward more biologically realistic models. When optimized with back-propagation (both the 1 × 1 CNNs and the NMF modules), we show that these networks exhibit performances on benchmark data sets that can exceed the values of pure CNNs with the same architecture.

Furthermore, another aspect of the NMF framework is its potential compatibility with spiking neural networks. Since the iterative updates of the NMF modules can be naturally mapped to event-based computations, the model can, in principle, be converted into a spiking implementation Rotermund and Pawelzik, (2019). Spiking neural networks are currently being actively investigated due to their unique properties, such as energy efficiency and their ability to perform online learning in dynamic environments (Pfeiffer and Pfeil, 2018; Lobo et al., 2020). This connection highlights an additional avenue where NMF-based architectures may provide insights into biologically plausible and resource-efficient neural computation.

Lastly, it is important to note that the primary goal of this work is not to establish a new state-of-the-art model in image classification. Instead, we aim to demonstrate that biologically inspired constraints—such as positive long-range interactions—can be integrated into deep neural networks while maintaining competitive performance at a comparable architectural scale.

## 2 Methods

### 2.1 Non-negative matrix factorization

Non-negative Matrix Factorization (NMF) (Lee and Seung, 1999, 2000) is a technique used to decompose a non-negative matrix *X* of *M* input vectors into two lower-dimensional non-negative matrices *W* and *H*, such that *X*≈*WH*, where $X\in {\mathbb{R}}_{+}^{M\times S}$, $W\in {\mathbb{R}}_{+}^{S\times I}$, and $H\in {\mathbb{R}}_{+}^{M\times I}$. The goal is to minimize the discrepancy between *X* and the product *WH* while ensuring that both *W* and *H* remain non-negative. It is often expressed as:


(1) $$\begin{array}{l} \underset{W,H}{\min}||X-WH||\;\text{subject to }{\left(W\right)}_{sj}=:W_{sj}\geq 0,{\left(H\right)}_{\mu j}=:h_{\mu_{j}}\geq 0 \end{array}$$

minimizing the Kullback-Leibler divergence defined as:


(2) $$\begin{array}{l} D\left(X||WH\right)=\underset{\mu ,s}{\sum }X_{\mu s}\ln\frac{X_{\mu s}}{\sum_{j}W_{sj}h_{\mu_{j}}} \end{array}$$

leads to the following multiplicative update rules for *W* and *H* (Lee and Seung, 2000):


(3) $$\begin{array}{l} h_{\mu i}\leftarrow h_{\mu i}\underset{s}{\sum }\frac{W_{si}X_{\mu s}}{\sum_{j}W_{sj}h_{\mu j}} \end{array}$$

(4) $$\begin{array}{l} W_{si}\leftarrow W_{si}\underset{\mu }{\sum }\frac{h_{\mu i}X_{\mu s}}{\sum_{j}W_{sj}h_{\mu j}} \end{array}$$

(5) $$\begin{array}{l} W_{si}\leftarrow \frac{W_{si}}{\sum_{j}W_{ji}} \end{array}$$

### 2.2 Deep non-negative matrix factorization in a neural network

In this work, we extend NMF to a deep learning setting (Chen et al., 2022) by integrating it into a network architecture. Specifically, we treat the factorized matrices *W* and *H* as components of a neural network layer. The matrix *W* is used as the weight matrix of the layer, while the matrix *H* represents the activation values (neuron outputs) of the layer.

The challenge lies in adapting the unsupervised nature of NMF (Lee and Seung, 1999) for use in a multi-layer supervised context, such as classification, where the learned weights must optimize a specific task-related objective function (Ciampiconi et al., 2023; Tian et al., 2022). In classical NMF, both *W* and *H* are updated iteratively using, for example, multiplicative update rules, following the Expectation-Maximization algorithm, to minimize the factorization error. However, when applying NMF within a neural network, directly updating *W* in an unsupervised manner can lead to weights that are not aligned with the task objective (e.g., classification loss). To address this, we decouple the update process for *W* from the factorization step.

Instead of updating *W* using the NMF update rules, we keep *W* fixed during the forward pass, using it to calculate the activations *H* for each layer. The neuron values at each iteration, on the other hand, follow a similar approach to the NMF update rule. For one pattern, the general update rule for *h* in a hidden layer at each iteration *t* is formulated as:


(6) $$\begin{array}{l} h_{i}\left(t\right)=h_{i}\left(t-1\right)+\varepsilon h_{i}\left(t-1\right)\left(\underset{s}{\sum }\frac{X_{s}W_{s,i}}{\sum_{i}W_{s,i}h_{i}\left(t-1\right)}-1\right) \end{array}$$

Where *X*_*s*_ denotes the input and *W*_*s, i*_ is the weight matrix. Here we omitted the index μ, representing different samples of data, for simplicity. Unless said otherwise, ε is set to 1, which leads to an equation similar to Equation 3. During each forward pass at each layer, we first initialize *h* values to $h_{i}\left(0\right)=\frac{1}{I}$, where *I* is the number of neurons, and we repeat the update rule (Equation 6) for *N* times until getting the output values of the layer.

In this setting, the factorization is applied repeatedly to the input activations during each forward pass, where the iterative update of *H* is part of the network dynamics. It is worth noting that this differs fundamentally from approaches such as Low-Rank Adaptation (LoRA) (Cer et al., 2017) that decompose weight matrices once for parameter reduction or fine-tuning; in our case, the decomposition operates on input representations and is tightly coupled with the learning dynamics of the entire network.

### 2.3 Approximated back-propagation

A significant practical challenge in implementing NMF-based neural architectures is the computational overhead of back-propagation through iterative steps. Conventional NMF requires *N* iterations (*N* usually ranging from 15 to 100 iterations) in the forward pass, and automatic differentiation frameworks like PyTorch must store gradients for each iteration to compute the backward pass accurately. This creates substantial memory requirements and computational bottlenecks, especially for deep networks or large-scale applications.

We address this limitation by using an efficient approximation to the back-propagation procedure that requires only a single step, eliminating the need to save and back-propagate through all intermediate iterations performed during the forward pass. This method, introduced in Rotermund and Pawelzik (2019), dramatically reduces both memory consumption and computation time while maintaining comparable accuracy to full back-propagation through all iterations. For convenience, we here only sketch the basic idea underlying this approach but refer to (Rotermund and Pawelzik 2019) for the detailed derivations. Back-propagation requires the partial derivatives $\frac{\partial h_{i}}{\partial x_{s}}$ and $\frac{\partial h_{i}}{\partial W_{s,j}}$. These derivatives can be obtained from the h-dynamics for a single pattern *x*:


(7) $$\begin{array}{l} h_{i}^{\prime }=h_{i}+\varepsilon h_{i}\left(\underset{s}{\sum }\frac{x_{s}W_{s,i}}{R_{s}}-1\right) \end{array}$$

(8) $$\begin{array}{ll} = & h_{i}+\delta h_{i}, \end{array}$$

where $R_{s}:=\underset{i}{\sum }W_{s,i}h_{i}$.

If we would change *x*→*x*+Δ*x* we would obtain a deterministic change of the output in one step of this dynamics:


(9) $$\begin{array}{l} h_{i}^{\prime }=h_{i}+\varepsilon h_{i}\left(\underset{s}{\sum }\frac{\left(x_{s}+\Delta x_{s}\right)W_{s,i}}{R_{s}}-1\right) \end{array}$$

(10) $$\begin{array}{l} =h_{i}+\varepsilon h_{i}\left(\underset{s}{\sum }\frac{x_{s}W_{s,i}}{R_{s}}+\underset{s}{\sum }\frac{\Delta x_{s}W_{s,i}}{R_{s}}-1\right) \end{array}$$

(11) $$\begin{array}{l} =h_{i}+\delta h_{i}+\Delta h_{i}. \end{array}$$

That is, we now have the changes of the original δ*h* depending on changes of the input Δ*x*:


(12) $$\begin{array}{l} \Delta h_{i}=\varepsilon h_{i}\left(\underset{s}{\sum }\frac{\Delta x_{s}W_{s,i}}{R_{s}}\right). \end{array}$$

This formula preserves normalization of *h* since $\underset{i}{\sum }\Delta h_{i}=0$. By comparing with the total differential, we obtain


(13) $$\begin{array}{lll} \frac{\partial h_{i}^{\prime }}{\partial x_{s}} & \propto & h_{i}\frac{W_{s,i}}{R_{s}}. \end{array}$$

Following the same logic, we have


(14) $$\begin{array}{l} h_{i}^{'}=h_{i}+\varepsilon \left(\sum_{s}\frac{x_{s}(W_{s,i}+\Delta W_{s,i})}{\sum_{j}\left(W_{s,j}+\Delta W_{s,j}\right)h_{j}}-1\right)\\ \;≃h_{i}+\delta h_{i}+\varepsilon h_{i}(\sum_{s}\frac{x_{s}(W_{s,i}+\Delta W_{s,i})}{R_{s}} \end{array}$$

(15) $$\;-\sum_{s}\frac{x_{s}(W_{s,i}+\Delta W_{s,i})(\sum_{j}\Delta W_{s,j}h_{j})}{R_{s}^{2}})$$

(16) $$\approx h_{i}+\delta h_{i}+\varepsilon h_{i}\left(\sum_{s}\frac{x_{s}\Delta W_{s,i}}{R_{s}}-\sum_{s}\frac{x_{s}(W_{s,i})(\sum_{j}\Delta W_{s,j}h_{j})}{R_{s}^{2}}\right).$$

which (again via the total differential) leads to


(17) $$\begin{array}{l} \frac{\partial h_{i}^{\prime }}{\partial W_{s,j}}=h_{i}\frac{x_{s}}{R_{s}}\left(\delta_{i,j}-\frac{W_{s,i}h_{j}}{R_{s}}\right). \end{array}$$

**Figure 1:**
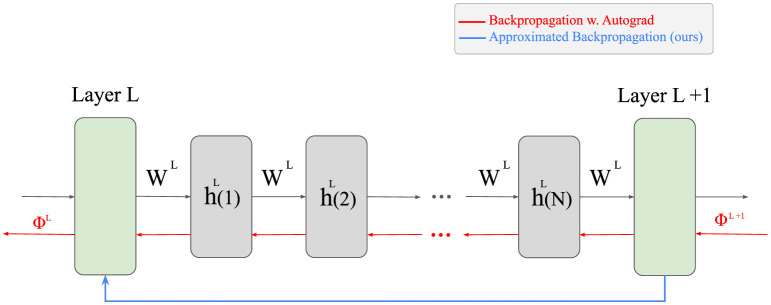
The difference between our approximative approach and the naive back-propagation. Since NMF is an iterative algorithm, the output of each layer is computed after several iterations of the update rule. To apply the vanilla back-propagation, all these intermediate steps are required to be saved to the memory during the forward pass, which is time- and memory-inefficient. Instead, our proposed approximated back-propagation can compute the corresponding error of a lower layer in one step, only utilizing the output of the layer.

These derivatives are then used to change the weights according to


(18) $$\begin{array}{l} \delta \omega_{si}=\frac{h_{i}X_{s}}{{\left(R_{s}\right)}^{2}}\left(\Phi_{i}^{L+1}R_{s}-\underset{j}{\sum }W_{sj}h_{j}\Phi_{j}^{L+1}\right) \end{array}$$

where


(19) $$\begin{array}{l} \Phi_{s}^{L}=\underset{i}{\sum }\Phi_{i}^{L+1}\frac{W_{si}h_{i}}{\sum_{j}W_{sj}h_{j}} \end{array}$$

is the back-propagated error $\Phi_{i}^{L+1}$. Applying this method only to the final states *h*(*N*) of each layer is provably sufficient. Importantly, all layers in the network are optimized jointly through back-propagation; the approximation only concerns the iterative updates within each NMF module, not a restriction to optimizing the last layer. Figure 1 illustrates how this approach reduces the amount of computations.

#### 2.3.1 Updating the weight matrix

To make the weight matrix *W* more suitable for classification, we update *W* through back-propagation using an optimizer (e.g., Adam). This ensures that *W* is optimized based on the task's objective function rather than simply minimizing the reconstruction error from NMF. However, a challenge arises as gradient-based updates do not inherently preserve the non-negativity and normalization properties of *W*. Since NMF requires that *W* remains non-negative and normalized, we cannot directly update *W* with the raw gradient values. Instead, we introduce a trainable auxiliary matrix *U*, which has the same dimensions as *W*, and at each network update, the optimizer will update *U* using the error calculated in the Equation 18. Based on this parameter, during each forward pass, the weight *W*_*s, i*_ is obtained based on:


(20) $$\begin{array}{l} W_{s,i}=\frac{\left|U_{s,i}\right|}{\sum_{k}^{S}|U_{k,i}|} \end{array}$$

which applies two main transformations:

1. **Non-negativity constraint**: We enforce non-negativity by setting *W* = |*U*|, where |*U*| represents the element-wise absolute value of *U*.
2. **Normalization**: We normalize each row of *W* to ensure the sum of each row is equal to 1, ensuring that *W* remains a valid factorization matrix.

The transformation ensures that the weight matrix *W* retains the necessary properties for NMF while still being adaptable for learning tasks.

**Figure 2:**
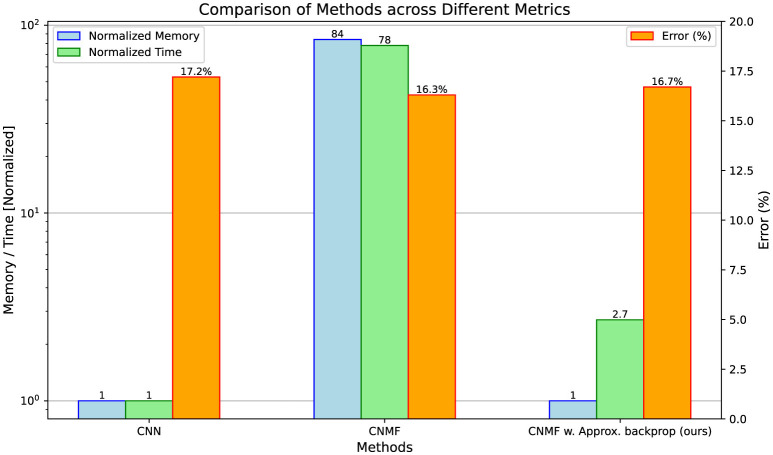
Prediction error and computational cost during the back-propagation between CNN, NMF, and NMF with approximate back-propagation (ours). The comparison spans three metrics: back-propagation memory consumption **(left)**, back-propagation computation time **(middle)**, and classification error **(right)**. Memory and time values are shown relative to the CNN baseline.

Our empirical evaluation confirms the computational efficiency of the proposed approximate back-propagation (BP) method while maintaining performance. Figure 2 compares three architectures: a normal convolutional network, an NMF-based network with full BP via Torch Autograd, and our NMF network with approximate BP. While the standard NMF implementation shows considerable computational costs, requiring significantly more memory and time compared to the CNN baseline, our approximation method dramatically reduces these overheads. Specifically, while achieving comparable classification accuracy to both baseline models, our approximate BP approach maintains the same memory footprint as the CNN model while operating the BP ≈29 times faster than the standard NMF.

These results demonstrate that our approximation strategy successfully addresses the primary computational bottleneck of NMF-based networks while preserving their advantages. This computational innovation makes NMF-based neural architectures more practical for real-world applications, allowing us to leverage their biological plausibility advantages without prohibitive computational costs.

### 2.4 Proposed methods

#### 2.4.1 Convolutional NMF

In conventional NMF (Lee and Seung, 2000), the input is reconstructed using a linear transformation of the latent values, implemented through regular matrix multiplication, which corresponds to a dense layer in a neural network architecture. However, this linear transformation can be replaced with other linear operations while preserving the core principles of NMF.

In our approach, we substitute the standard matrix multiplication with a convolution operation, resulting in Convolutional NMF (CNMF). This adaptation maintains the mathematical foundations of NMF while leveraging the spatial locality benefits of convolutions. As demonstrated in our previous work (Rotermund et al., 2023), CNMF can be effectively trained using back-propagation and exhibits remarkable noise robustness when compared to conventional CNNs.

While CNMF shows superior performance in noisy conditions, on clean data, it does not consistently outperform standard CNNs with comparable architectures. To address this limitation and further enhance the capabilities of our CNMF approach, we propose an extended architecture incorporating additional components as described in the following sections.

#### 2.4.2 1 × 1 convolutions

The non-negative constraint in NMF layers causes the network to represent data as a combination of basic building blocks (or “parts”) that are added together, rather than canceled out. This approach excels at identifying the key components within input data. However, because NMF is fundamentally a linear method, it struggles to capture complex patterns that involve non-linear relationships between features. Our proposed architecture combines NMF convolutional layers with a layer of a convolutional neural network with 1 × 1 kernels, providing several key advantages. The subsequent 1 × 1 convolutional layer, with its ability to use negative weights, remixes these features by allowing for subtraction and adjustment, which NMF alone cannot achieve since it can only add up contributions. This layer processes the data locally, providing detailed modulations of the more global patterns identified by the NMF layer.

**Figure 3:**
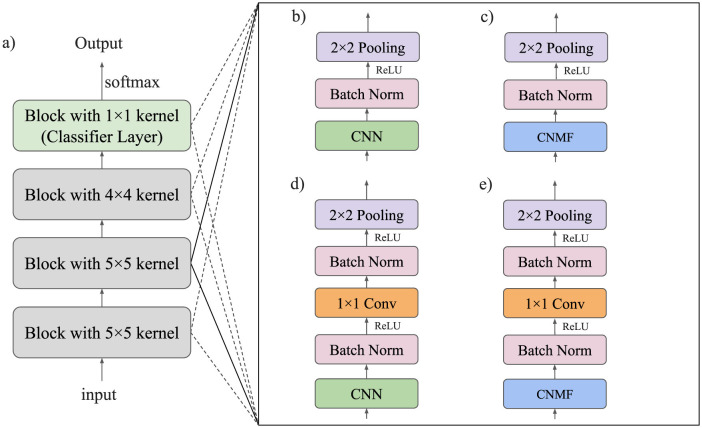
Model architecture of all investigated networks. **(a)** Overall model architecture. All three convolutional layers consist of one of the modules listed on the right. **(b)** Module used in the baseline CNN model. **(c)** Module used in the CNMF model. **(d)** Module used in the CNN + 1×1 Conv model. **(e)** Module used in the CNMF + 1×1 Conv model.

A diagram of this module is provided in Figure 3e. We compare this model to our previous CNMF module (Figure 3c) from Rotermund et al. (2023) and its corresponding CNN model (Figure 3b). We also compare this model to a similar CNN architecture shown in section (Figure 3d) of the figure.

### 2.5 Model architecture

The architecture of all proposed models consists of a sequence of four processing blocks as illustrated in Figure 3a. Each block incorporates either a CNN or CNMF module, which may be followed by a 1 × 1 convolutional layer for local feature mixing. After each layer, we apply batch normalization followed by ReLU activation.

**Figure 4:**
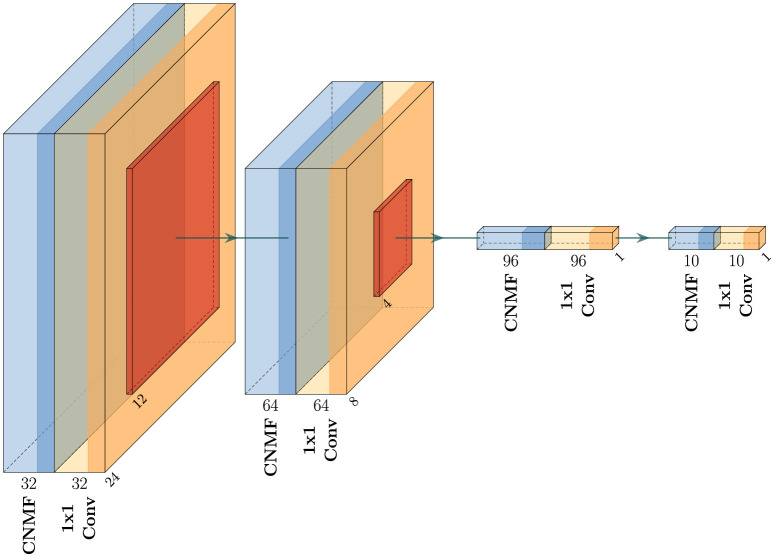
Network architecture of the proposed method for the CNMF + 1 × 1 Convolution. The network consists of four sequential blocks, each containing a CNMF module followed by a 1 × 1 convolutional layer. The architecture progressively reduces spatial dimensions from 28 × 28 in the input to 1 × 1 while transforming feature channels (32 → 64 → 96 → 10 → output). The output of the last 1 × 1 convolutional layer is used for the classification. For simplicity, activations and batch normalization layers are omitted from the figure.

Figure 4 provides a detailed representation of our CNMF + 1 × 1 convolution implementation (shown in Figure 3e), highlighting how the architecture progressively transforms the input through successive layers. For optimization purposes, we omit batch normalization in the final two blocks.

#### 2.5.1 Loss function

To optimize our models, we employed a composite loss function that combines cross-entropy (CE) loss with mean squared error (MSE). The loss function is defined as:


(21) $$\begin{array}{l} L=-\underset{i}{\sum }y_{i}\log\left({ŷ}_{i}\right)+\alpha \underset{i}{\sum }{\left(y_{i}-{ŷ}_{i}\right)}^{2} \end{array}$$

where *y*_*i*_ represents the true label (one-hot encoded), ŷ_*i*_ represents the predicted probability distribution over classes, and α = 0.5 is a weighting factor that balances the contribution of each component. While cross-entropy loss effectively optimizes for correct classification by heavily penalizing errors in the predicted class, it primarily focuses on the correct label and may not fully capture the relationship between incorrect predictions. By incorporating MSE with a smaller weight (α = 0.5), we introduce an additional regularizing term that considers the full distribution of predictions across all classes. This combined loss function led to consistent performance improvements across all model architectures in our experiments.

### 2.6 Implementation

We evaluated the performance of our proposed model on the CIFAR-10 dataset, comparing it to a CNN and NMF model similar to those described in Rotermund et al. (2023). All models were trained using the Adam optimizer with an initial learning rate of 0.001. To ensure optimal convergence, we implemented a learning rate reduction strategy where the rate was decreased by a factor of 10 whenever the validation loss plateaued for 10 consecutive epochs. The training was terminated either when the learning rate dropped below 10^−9^ or when reaching the maximum limit of 500 epochs, whichever occurred first. For data augmentation, we applied random horizontal flips and color jitter to the training images. We also apply a random crop on the input image from 32 × 32 to 28 × 28. All hyperparameters were kept consistent across different model architectures to ensure a fair comparison. The models were implemented in PyTorch and trained on an NVIDIA GeForce RTX 4090 GPU.

The source containing all models and training setups can be found under: https://github.com/mahbodnr/deep_nmf.

## 3 Results

**Figure 5:**
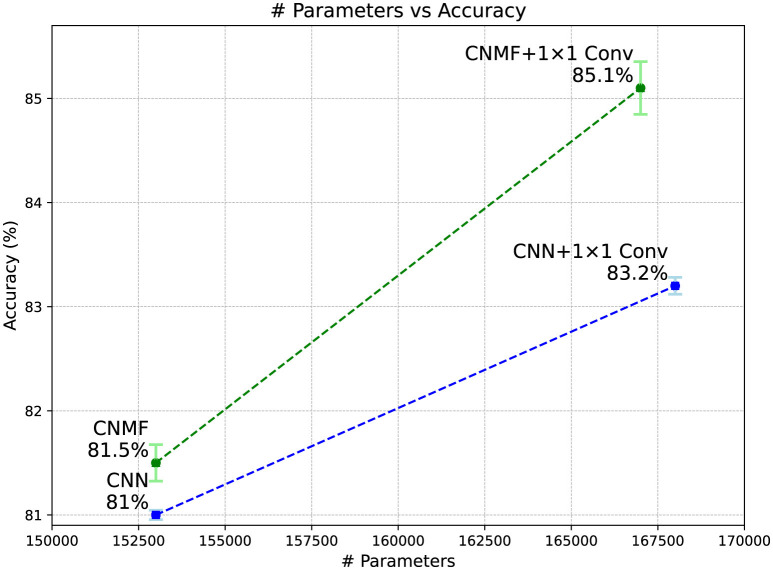
Classification accuracy of models on the CIFAR-10 dataset. Error bars represent the standard deviation across five models, and the plotted values correspond to their average performance over different random initializations.

Figure 5 displays the classification accuracy achieved by all models on the CIFAR-10 dataset alongside their parameter counts. The results demonstrate that augmenting the CNMF model with 1 × 1 convolutions substantially improves performance, allowing it to significantly outperform the baseline CNN model of comparable architecture and size.

### 3.1 Effect of NMF compared to CNN in the network

To investigate whether the performance improvements in our model stem primarily from the CNN components or if the NMF modules play a crucial role, we conducted an extensive analysis across different model configurations. We generated 100 different model variants by systematically adjusting the network architecture in two ways: first, by scaling the number of neurons in each layer (multiplying by factors of 2, 4, and 8), and second, by varying the number of groups in both NMF and CNN layers (using 1, 2, 4, 8, and 16 groups). When we increase the number of groups in a layer, we divide its channels into separate groups that process the input independently, thereby reducing the number of parameters while maintaining the same input and output dimensions. This approach allowed us to explore models with different ratios of NMF to CNN parameters while maintaining the overall architectural structure.

**Figure 6:**
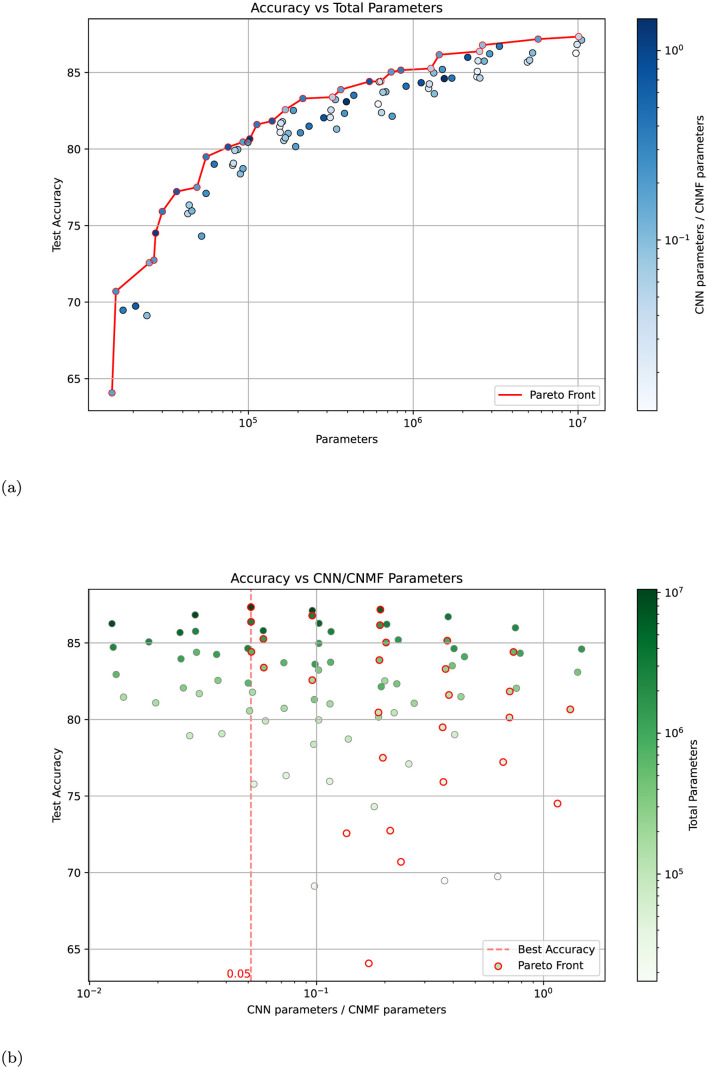
Analysis of model performance across different parameter distributions between CNN and NMF components. **(a)** Test accuracy vs. total parameter count for 100 model variants, with color intensity indicating the ratio of CNN to NMF parameters (darker blue = higher CNN/CNMF ratio). The red line shows the Pareto front of optimal-performing models. **(b)** Test accuracy vs. CNN/CNMF parameter ratio, with color intensity indicating total parameter count (darker green = more parameters). The red dashed line marks the ratio achieving the highest accuracy. Models belonging to the Pareto front are indicated with a red edge. Both plots were generated by varying the number of neurons (×1, ×2, ×4, ×8) and groups (1, 2, 4, 8, 16) in the base architecture.

The results of this analysis are presented in Figure 6. Figure 6a shows model accuracy vs. total parameter count, with the color intensity indicating the ratio of CNN to NMF parameters. The Pareto front, which represents the best-performing models for a given parameter budget, shows no systematic bias toward models with higher CNN parameter ratios. This suggests that simply increasing the proportion of CNN parameters does not lead to optimal performance. Figure 6b provides a complementary view, plotting accuracy against the ratio of CNN to NMF parameters, with color intensity representing the total parameter count. The distribution of high-performing models appears roughly symmetric around a balanced ratio, indicating that the best results are achieved when neither component dominates the network. Notably, the highest accuracy (indicated by the red dashed line) is achieved with a nearly balanced distribution of parameters between CNN and NMF components.

These findings strongly suggest that the NMF modules are not merely a simple non-linear transformation block but are essential contributors to the network's performance. The optimal performance achieved with a balanced parameter distribution indicates a synergistic relationship between the NMF and CNN components, where each plays a crucial and complementary role in the network's processing capabilities.

## 4 Discussion and limitations

Our work demonstrates that incorporating biologically-inspired computational principles into deep neural networks can enhance their performance while maintaining biological plausibility. By combining NMF with local mixing through 1 × 1 convolutions, we achieved classification accuracy that matches or exceeds standard CNNs on the CIFAR-10 dataset, while preserving key biological constraints such as positive long-range interactions and local inhibitory processing. Further experiments on the FashionMNIST (Xiao et al., 2017) and CIFAR-100 datasets confirm these conclusions. A report for these experiments can be found in Appendix A.

It is worth mentioning that there remains a clear performance gap between the models evaluated in this work and the current state of the art. For instance, EfficientNetV2 models have been reported to achieve up to 99.1% accuracy on CIFAR-10 (Tan and Le, 2021). However, the main goal of our study is not to compete with such models in terms of benchmark accuracy. Rather, our intention is to demonstrate that biologically inspired constraints, such as positive long-range interactions, can be incorporated into deep learning architectures while still achieving competitive performance compared to conventional models of similar scale. Finally, it should be emphasized that state-of-the-art approaches are typically trained at a much larger scale and under substantially different conditions. For example, the EfficientNetV2-L model contains 121M parameters (compared to < 170k parameters in our proposed models) and benefits from extensive preprocessing, aggressive data augmentation, and pre-training on much larger datasets. Exploring whether our biologically grounded models can remain competitive under such large-scale settings is an important question, but it lies beyond the scope of the present work.

### 4.1 Bridging biological and artificial neural computation

A fundamental distinction between biological neural computation and artificial neural networks lies in their computational dynamics. In biological systems, most neural processing occurs through implicit layers with complex recurrent interactions and iterative refinement of neural responses. This is evident in the cortical microcircuits, where information is processed through multiple recursive loops between different neural populations before a stable representation emerges. In contrast, artificial neural networks predominantly rely on explicit feedforward computation, which, while computationally efficient, diverges significantly from biological reality.

Our approach bridges this gap by implementing NMF as an implicit layer that converges through iterative updates, more closely mimicking biological neural dynamics. While conventional feedforward networks like CNNs have dominated deep learning due to their computational efficiency and straightforward optimization, our results suggest that biologically inspired implicit computation can be equally effective when properly implemented. To make this happen, the key innovation in our work is the combination of iterative NMF processing with local feature mixing, which parallels the interaction between long-range excitatory connections and local inhibitory circuits in cortical processing.

Moreover, the demonstrated benefits of interleaving non-negative convolution with local mixing can be directly transferred to the Spike-by-Spike (SbS) model that we have developed in previous work (Rotermund and Pawelzik, 2019). SbS implements the same NMF principles in a spike-based framework, which is both more biologically plausible and potentially far more energy-efficient. Moreover, SbS has already been shown to maintain high robustness under noise, and we expect that integrating the present CNMF insights will further enhance its performance (Nevarez et al., 2021; Najafi et al., 2023). Exploring this extension will be a focus of our future work.

### 4.2 Analysis of feature selection in NMF networks

To understand the limitations of hierarchical NMF networks trained with unsupervised learning rules and subsequently fine-tuned for classification, it is helpful to decompose the input data into five distinct components:

- CD: Class-relevant Dominant statistical features.
- CN: Class-relevant Non-dominant statistical features.
- UD: class-Unrelated Dominant statistical features.
- UN: class-Unrelated Non-dominant statistical features.
- N: Noise.

The key distinction between back-propagation and NMF's local learning lies in their feature selection characteristics. Back-propagation, guided by the classification objective, effectively extracts both dominant and non-dominant class-relevant features (CD and CN). In contrast, NMF's unsupervised learning rule, which optimizes for reconstruction based on statistical prominence, primarily captures dominant features regardless of their relevance to classification (CD and UD).

This fundamental difference creates a critical issue: when using NMF's local learning rules instead of back-propagation, the non-dominant but class-relevant features (CN) are progressively filtered out as information flows through the network layers. By the time the signal reaches the output layer, these crucial classification features have been lost, despite their importance for the discrimination task. This explains the reduced classification performance observed in networks trained with NMF's unsupervised learning rules. For example, a model with a similar architecture to our CNMF model will achieve 32% accuracy when the NMF modules are trained only based on the local learning rule on the same task (as opposed to the 81.5% that is achieved by using backpropagation).

This analysis highlights why our approach of using supervised gradient descent to update the weights while maintaining NMF's non-negativity constraints provides superior classification performance.

## Data Availability

Publicly available datasets were analyzed in this study. This data can be found here: https://www.cs.toronto.edu/~kriz/cifar.html.

## References

[B1] Callaway E. M. (1998). Local circuits in primary visual cortex of the macaque monkey. Annu. Rev. Neurosci. 21, 47–74. 10.1146/annurev.neuro.21.1.47 9530491

[B2] Cer D. Diab M. Agirre E. Lopez-Gazpio I. Specia L. (2017). “Semeval-2017 task 1: semantic textual similarity multilingual and crosslingual focused evaluation,” in Proceedings of the 11th International Workshop on Semantic Evaluation (SemEval-2017) (Association for Computational Linguistics). 10.18653/v1/S17-2001

[B3] Chen W.-S. Zeng Q. Pan B. (2022). A survey of deep nonnegative matrix factorization. Neurocomputing 491, 305–320. 10.1016/j.neucom.2021.08.152

[B4] Ciampiconi L. Elwood A. Leonardi M. Mohamed A. Rozza A. (2023). A survey and taxonomy of loss functions in machine learning. arXiv preprint arXiv:2301.05579.

[B5] Fukushima K. (1980). Neocognitron: a self-organizing neural network model for a mechanism of pattern recognition unaffected by shift in position. Biol. Cybern. 36, 193–202. 10.1007/BF00344251 7370364

[B6] Geng Z. Guo M.-H. Chen H. Li X. Wei K. Lin Z. (2021). Is attention better than matrix decomposition? *arXiv preprint arXiv:2109.04553*.

[B7] Hoyer P. O. (2003). Modeling receptive fields with non-negative sparse coding. Neurocomputing 52, 547–552. 10.1016/S0925-2312(02)00782-8

[B8] Hubel D. H. Wiesel T. N. (1962). Receptive fields, binocular interaction and functional architecture in the cat's visual cortex. J. Physiol. 160, 106–154. 10.1113/jphysiol.1962.sp006837 14449617 PMC1359523

[B9] Lecun Y. Bottou L. Bengio Y. Haffner P. (1998). Gradient-based learning applied to document recognition. Proc. IEEE 86, 2278–2324. 10.1109/5.726791

[B10] Lee D. Seung H. S. (2000). “Algorithms for non-negative matrix factorization,” in Advances in Neural Information Processing Systems, 13.

[B11] Lee D. D. Seung H. S. (1999). Learning the parts of objects by non-negative matrix factorization. Nature 401, 788–791. 10.1038/44565 10548103

[B12] Lobo J. L. Del Ser J. Bifet A. Kasabov N. (2020). Spiking neural networks and online learning: an overview and perspectives. Neural Netw. 121, 88–100. 10.1016/j.neunet.2019.09.004 31536902

[B13] Martin K. (1991). A functional microcircuit for cat visual cortex. J. Physiol. 440, 735–769. 10.1113/jphysiol.1991.sp018733 1666655 PMC1180177

[B14] Najafi A. Rotermund D. Najafi A. Pawelzik K. R. Garcia-Ortiz A. (2023). “Empirical analysis of full-system approximation on non-spiking and spiking neural networks,” in 2023 12th International Conference on Modern Circuits and Systems Technologies (MOCAST), 1–5. 10.1109/MOCAST57943.2023.10176919

[B15] Nevarez Y. Rotermund D. Pawelzik K. R. Garcia-Ortiz A. (2021). Accelerating spike-by-spike neural networks on fpga with hybrid custom floating-point and logarithmic dot-product approximation. IEEE Access 9, 80603–80620. 10.1109/ACCESS.2021.3085216

[B16] Olshausen B. A. Field D. J. (1996). Emergence of simple-cell receptive field properties by learning a sparse code for natural images. Nature 381, 607–609. 10.1038/381607a0 8637596

[B17] Olshausen B. A. Field D. J. (2006). What is the other 85 percent of v1 doing. L. van Hemmen, and T. Sejnowski (Eds.) 23, 182–211. 10.1093/acprof:oso/9780195148220.003.0010

[B18] Pfeiffer M. Pfeil T. (2018). Deep learning with spiking neurons: opportunities and challenges. Front. Neurosci. 12:409662. 10.3389/fnins.2018.00774 30410432 PMC6209684

[B19] Rotermund D. Garcia-Ortiz A. Pawelzik K. R. (2023). Competitive performance and superior noise robustness of a non-negative deep convolutional spiking network. bioRxiv, 2023-04. 10.1101/2023.04.22.537923

[B20] Rotermund D. Pawelzik K. R. (2019). Back-propagation learning in deep spike-by-spike networks. Front. Comput. Neurosci. 13:55. 10.3389/fncom.2019.00055 31456677 PMC6700320

[B21] Strata P. Harvey R. (1999). Dale's principle. Brain Res. Bull. 50, 349–350. 10.1016/S0361-9230(99)00100-8 10643431

[B22] Tan M. Le Q. V. (2021). Efficientnetv2: Smaller models and faster training. arXiv preprint arXiv:2104.00298.

[B23] Tian Y. Su D. Lauria S. Liu X. (2022). Recent advances on loss functions in deep learning for computer vision. Neurocomputing 497, 129–158. 10.1016/j.neucom.2022.04.127

[B24] Xiao H. Rasul K. Vollgraf R. (2017). Fashion-mnist: a novel image dataset for benchmarking machine learning algorithms. arXiv preprint arXiv:1708.07747.

[B25] Yang W. Carrasquillo Y. Hooks B. M. Nerbonne J. M. Burkhalter A. (2013). Distinct balance of excitation and inhibition in an interareal feedforward and feedback circuit of mouse visual cortex. J. Neurosci. 33, 17373–17384. 10.1523/JNEUROSCI.2515-13.2013 24174670 PMC3812505

