# Supplementary material for: Interleaving cortex-analog mixing improves deep non-negative matrix factorization networks
Source: Front Comput Neurosci. 2025 Nov 5;19:1692418. doi: 10.3389/fncom.2025.1692418 (PMC12626930; doi:10.3389/fncom.2025.1692418)
Supplement: Supplementary file 1 [file Data_Sheet_1.pdf]

Supplementary Material

1 APPENDIX A

We have evaluated the performance of our models on the FashionMNIST and CIFAR-100 datasets. The results are presented in the following tables. Training setups are similar to section 2.6. The proposed models are also compared to VGG (Simonyan and Zisserman, 2014) and ViT (Dosovitskiy et al., 2020) models.

For VGG8, we have used several models suggested in (Rambhatla et al., 2021) that are in the same scale of number of parameters as our proposed models. We have also introduced a similar model with batch normalization. For ViT model, we used the same model as suggested in (Dosovitskiy et al., 2020) with smaller dimensions to match the scale of the number of parameters of our proposed models.

1.1 FashionMNIST

Model	Parameters	Accuracy
CNN	153k	84
CNMF	153k	86
CNN+1×1 Conv	167k	91
CNMF+1×1 Conv	167k	91
VGG	184k	92
VGG+norm	184k	85
ViT	411k	89

1.2 CIFAR 100

The following tables summarize the performance of our models on the CIFAR-100 dataset. The VGG and ViT models are similar to the ones used in the FashionMNIST experiment.

Model	Parameters	Accuracy
CNMF	153k	23
CNN	153k	36
CNN+1×1 Conv	167k	42
CNMF+1×1 Conv	167k	48
VGG+norm	184k	37
ViT	411k	43

1.2.1 Different scales

We have also evaluated the performance of our models on the CIFAR-100 dataset with different scales. The results are presented in the following tables. The VGG8 small model is similar to the VGG model used in the FashionMNIST experiment. The VGG8 medium and large models have, respectively, the following convolutional hidden dimensions: [16, 45, 90, 181, 307] and [18, 50, 101, 202, 344].

Model	Parameters	Accuracy
CNMF+1×1 Conv	186k	48
CNMF+1×1 Conv ×2	360k	54
CNMF+1×1 Conv ×4	707k	58
CNMF+1×1 Conv ×8	1.4M	60
ViT 3 Layers	425k	33
ViT 6 Layers	823k	34
ViT 9 Layers	1.2M	44
VGG8 Small	405k	47
VGG8 Medium	761k	50
VGG8 Large	939k	51

The following figure summarizes the table for the CIFAR-100 dataset.

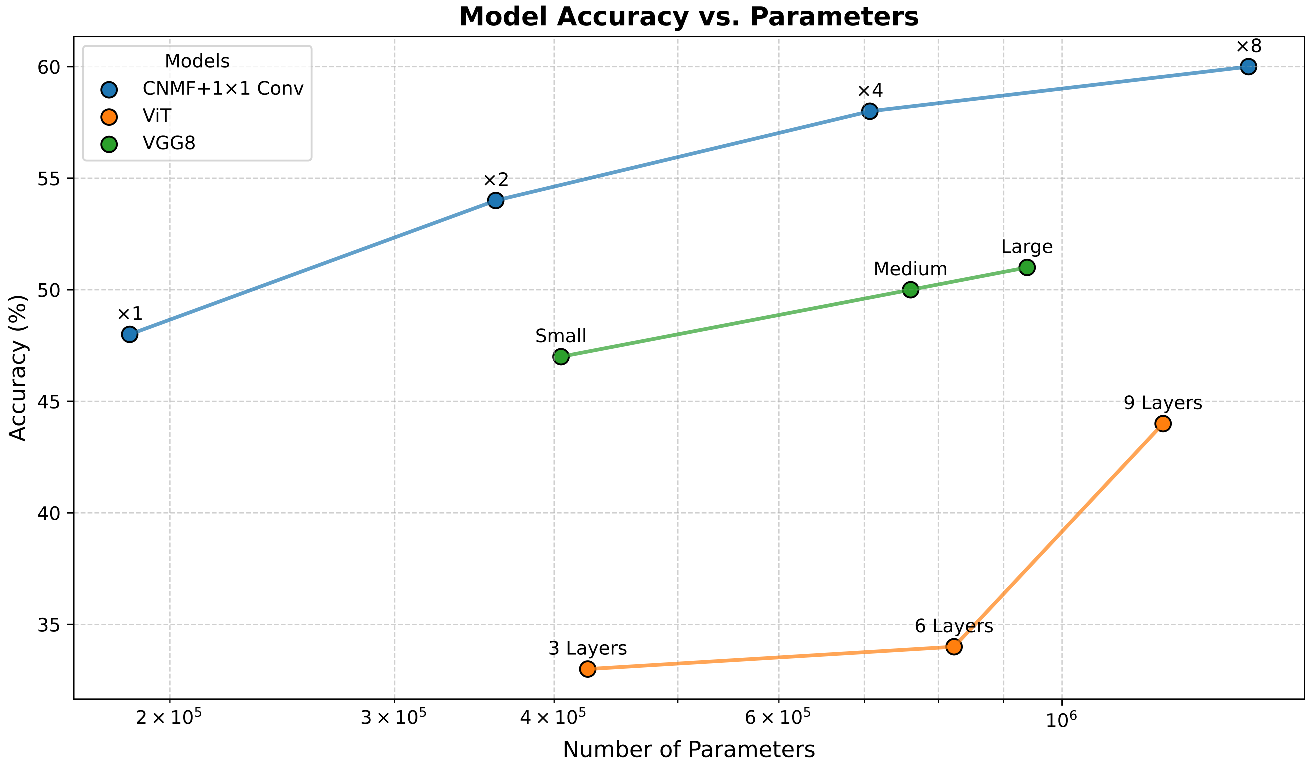

Figure S1. Comparison of model performance on the CIFAR-100 dataset with different scales.

REFERENCES

Dosovitskiy, A., Beyer, L., Kolesnikov, A., Weissenborn, D., Zhai, X., Unterthiner, T., et al. (2020). An image is worth 16x16 words: Transformers for image recognition at scale doi:10.48550/ARXIV.2010.11929

Rambhatla, S. S., Jones, M., and Chellappa, R. (2021). To boost or not to boost: On the limits of boosted neural networks doi:10.48550/ARXIV.2107.13600

Simonyan, K. and Zisserman, A. (2014). Very deep convolutional networks for large-scale image recognition doi:10.48550/ARXIV.1409.1556
